# Association between *OLR1* K167N SNP and Intima Media Thickness of the Common Carotid Artery in the General Population

**DOI:** 10.1371/journal.pone.0031086

**Published:** 2012-02-09

**Authors:** Irene Marta Predazzi, Giuseppe Danilo Norata, Lucia Vecchione, Katia Garlaschelli, Francesca Amati, Liliana Grigore, Lucia Cutuli, Angela Pirillo, Simona Tramontana, Francesco Romeo, Giuseppe Novelli, Alberico Luigi Catapano

**Affiliations:** 1 Department of Biopathology and Diagnostic Imaging, Section of Medical Genetics, School of Medicine, Tor Vergata University, Rome, Italy; 2 Center for Human Genetics Resesarch, Vanderbilt University Medical Center, Nashville, Tennessee, United States of America; 3 Center for the Study of Atherosclerosis, Società Italiana Studio Aterosclerosi, Ospedale Bassini, Gorki, Cinisello Balsamo, Italy; 4 Department of Pharmacological Sciences, Università di Milano, Milan, Italy; 5 Multimedica IRCCS, S.S. Giovanni; 6 Department of Internal Medicine, University of Rome Tor Vergata, Rome, Italy; 7 Ospedale San Pietro FBF, Rome, Italy; 8 National Agency for the Evaluation of Universities and Research, ANVUR, Rome, Italy; Innsbruck Medical University, Austria

## Abstract

**Background and Purpose:**

The lectin-like oxidised LDL receptor-1 (*OLR1*) gene encodes a scavenger receptor implicated in the pathogenesis of atherosclerosis. Although functional roles have been suggested for two variants, epidemiological studies on *OLR1* have been inconsistent.

*Methods* - We tested the association between the non-synonymous substitution K167N (rs11053646) and intima media thickness of the common carotid artery (CCA-IMT) in 2,141 samples from the Progression of Lesions in the Intima of the Carotid (PLIC) study (a prospective population-based study).

**Results:**

Significantly increased IMT was observed in male carriers of the minor C (N) allele compared to GC and GG (KN and KK) genotype. Functional analysis on macrophages suggested a decreased association to Ox-LDL in NN carriers compared to KN and KK carriers which is also associated with a reduced *OLR1* mRNA expression. Macrophages from NN carriers present also a specific inflammatory gene expression pattern compared to cells from KN and KK carriers.

**Conclusions:**

These data suggest that the 167N variant of LOX-1 receptor affects the atherogenic process in the carotid artery prior to evidence of disease through an inflammatory process.

## Introduction

Atherosclerosis is a complex disease. Endothelial dysfunction, activation and inflammation, proteolysis, apoptosis, platelet aggregation, thrombosis and angiogenesis are the processes involved in the phases of the disease [1]. The effects of environment on these phenotypes are also impacted by underlying genetic predisposition that may not impact all endo-phenotypes in the same way. The internalization of Ox-LDL has a critical effect in both endothelial dysfunction and inflammation [2]. This process is mediated by several scavenger receptors [3], including the lectin-like oxidized low-density lipoprotein receptor 1 (LOX-1) [4]. LOX-1 is mainly expressed in macrophages, endothelial, and smooth muscle cells. Its expression is induced by pro-inflammatory stimuli, such as shear stress, TNFα, LPS and infections [5], [6], [7]. LOX-1 is encoded by the *OLR1* gene, mapped to chromosome 12p13 [8]. A Single Nucleotide Polymorphisms (SNP) on exon 4, rs11053646 (G501C), leads to an amino acidic substitution (lysine to asparagine at position 167, K167N). Functional analyses suggested that a change on the positive isopotential surface determined by this variant could lead to a decreased binding and internalization of Ox-LDL [9].

Despite the evidence for a functional role of this polymorphism, results from epidemiological studies are equivocal [10], [11], [12], [13], [14], [15]. Of note, a gender specific association has been recently described between the C [N] allele and prevalence of carotid plaque in females of Dominican-Hispanic origin [16].

A direct association between circulating Ox-LDL and Intima Media Thickness (IMT) of the Common Carotid Artery has been demonstrated in previous studies [17], [18], [19]. Circulating Ox-LDL and IMT resulted inversely related to anti-OxLDL antibodies titulation, suggesting that immune response to Ox-LDL could have a protective role in the early phases of the disease [17], [18], [19].

Since the N allele of rs11053646 is associated to lower levels of Ox-LDL internalization [9], we tested whether this allele is associated to CCA-IMT in the Progression of Lesions in the Intima of the Carotid Artery (PLIC) study (a prospective population-based study representative of the population of Northern Milan, Italy).

In addition, we investigated functional effects in macrophages obtained from carriers of different genotypes to test the hypothesis that N allele could have a reduced receptor activity. If this is true, that should result in a less effective Ox-LDL binding and internalization and therefore display lower levels of *OLR1* RNA expression (as it is stimulated by Ox-LDL internalization itself).

## Methods

### Study sample

The use of human material in this study conforms to the principles outlined in the declaration of Helsinki. A cohort of 2,141 subjects attending the Atherosclerosis Centre in Bassini Hospital, Department of Pharmacological Sciences (University of Milan, Italy), was recruited for the PLIC study. This study has been previously widely described and the samples utilized in genetic studies [20], [21], [22], [23], [24], [25], [26].

### Genotyping

Genomic DNA was extracted using the Flexigene DNA kit (Qiagen, Milan, Italy) according to the manufacturer's instructions. SNP genotyping was performed through the Taqman Genotyping Assay (ID: C__22273024_10, Applied Biosystems, Foster City, CA) on a BioRad machine. One µL (10–200 ng) of DNA was analysed for genotyping.

A Genotype confirmation of ∼50 samples was obtained through Sanger sequencing on an ABI3130 machine (Methods S1).

### Statistical analysis

Group differences were determined by using Analysis of Variance (ANOVA) for continuous variables and chi-square analysis for categorical variables. Group differences with P<0.1 were considered as suggestive and P<0.05 was deemed as statistically significant. Plots were generated using Excel and data were analyzed using the SPSS Software (http://www.spss.it/) on a Windows Machine.

### Peripheral blood mononuclear cell (PBMC) isolation and culture

Peripheral blood mononuclear cells (PBMCs) were obtained from two subjects carrying the CC genotype (NN) and eight CG and GG subjects (KN and KK). These subjects were healthy and of comparable age, gender and blood lipid profiles. Blood samples diluted 1∶3 in phosphate-buffered saline (PBS; 15 mL, PH 7.4) were layered onto 4 mL of Ficoll-Hypaque (Amersham, Milan, Italy) and centrifuged at 300 g for 35 min. PBMCs were removed from the interface and washed twice (10 min, 300 g) in PBS before being counted.

PBMCs were re-suspended in RPMI supplemented with antibiotics and 10% serum bovine serum albumin and plated in 6-well plates and incubated for 1 1/2 hours at 37°C. Non-adherent cells were removed by rinses of PBS (4×). For addressing the association of Ox-LDL with macrophages, 1 week after the isolation, monocyte–derived macrophages were incubated with or without TNFα (10 ng/µL) for 18 h followed by Ox-LDL (6.25 or 12.5 µg/mL) for 1 h and then processed for FACS analysis.

### Expression analysis

Total RNA was extracted from circulating monocytes and from monocytes-derived macrophages cells according to manufacturer's Trizol protocol [27] and reverse transcription was performed as described [20]. Three µL of cDNA was amplified by real-time quantitative polymerase chain reaction (PCR) with 1× Sybr green universal PCR mastermix (Applied Biosystems, Foster City, CA). The specificity of the Sybr green fluorescence was tested by plotting fluorescence as a function of temperature to generate a melting curve of the amplicon. The melting peaks of the amplicons were as expected (data not shown). The primers used are reported in (Table S1). *HPTR1* was used as internal reference. Each sample was analyzed in duplicate using the Applies Biosystems 7000 machine and each experiment was replicated twice. The PCR amplification was related to a standard curve ranging from 10^−11^ to 10^−14^ mol/L.

### Flow cytometry

#### Isolation and modification of low density lipoproteins

LDLs (d = 1.019–1.063 g/mL) were isolated from fresh plasma of normolipidemic healthy volunteers by sequential ultracentrifugation [28]. Protein content was determined by the method of Lowry, using BSA as a standard [29]. Ox-LDL were generated with CuSO_4_ 5 µM as described [30].

#### Fluorescent labeling of lipoproteins

For lipid labeling, Ox-LDL were incubated with the fluorescent dye DiO (300 µg DiO/mg OxLDL protein) in PBS for 18 h at 4°C, passed over a PD10 column to remove unbound DiO, then centrifuged in a TL100 centrifuge at d = 1,063 g/mL for 2 h at 4°C. DiO-labeled lipoproteins were passed through a PD10 column and protein content was determined by the method of Lowry [29].

#### Cell-association studies

Cells were incubated at 37°C for 1 h with the indicated concentrations of Ox-LDL labeled with DiO. Cells were then washed three times with cold PBS, detached by scraping, fixed in 1% paraformaldehyde and immediately subjected to fluorescence flow cytometry using a FACScan (Becton Dickinson). For each sample 10,000 events were analyzed; data were processed using the CellQuest program (Becton Dickinson) [31].

## Results

The relative frequencies of the three *OLR1* genotypes are shown in Table 1. No deviation from Hardy Weinberg equilibrium was observed.

**Table 1:** Observed and expected frequencies of *OLR1* K167N polymorphism in the PLIC population and comparison with those reported in dbSNP (http://www.ncbi.nlm.nih.gov/projects/SNP/snp_ref.cgi?rs=11053646).

	KK	KN	NN	p-value
Observed frequency	1988 (87%)	283 (12%)	5 (0.02%)	0.12
Expected frequency	1992.4 (88%)	274.1 (12%)	9.4 (0.04%)	
dbSNP frequency CEU	75%	25%	0%	

The presence of the N allele was not associated with any of the cardiometabolic variables analysed (Table 2) and the increase in IMT observed was not statistically significant (0.64±0.12 mm, 0.66±0.11 mm and 0.69±0.07 for KK, KN and NN respectively, p = n.s). As a recent paper showed a gender related effect of the N allele [16], data were next analysed according to gender. The trend observed for systolic and diastolic blood pressures in the general population, was lost when stratifying by gender. However, in males, KK subjects showed a lower IMT compared to carriers of the N allele (NN and KN) [(0.66±0.13) mm and (0.69±0.13) mm respectively, p<0.05, Table 3]. No other phenotype was associated with this variant. Of note, no major phenotypes were observed in women. Furthermore, a second functional variation on *OLR1*, rs3736235 [32], was genotyped and no significant difference in cardiometabolic variables or IMT between carriers of the different genotypes was observed.

**Table 2:** K167N polymorphism in the PLIC population.

	K167N (rs11053646)		
	KK	KN+NN	p-value
Age (years)	54.33+/−0.260	54.43+/−0.680	ns
Systolic blood pressure (mmHg)	133.33+/−0.420	131.23+/−1.050	0.070
Diastolic blood pressure (mmHg)	82.98+/−0.220	81.85+/−0.600	0.095
Total cholesterol (mmol L)1)	221.77+/−0.890	220.07+/−2.400	ns
HDL cholesterol (mmol L)1)	56.13+/−0.340	54.07+/−0.810	ns
LDL cholesterol (mmol L)1)	144.205+/−0.820	144.717+/−2.180	ns
Triglycerides (mmol L)1)	108.11+/−1.420	105.58+/−3.280	ns
IMTm (mm)	0.645+/−0.003	0.658+/−0.007	0.080

**Table 3:** Statistical observations on the association between *OLR1* polymorphisms in males.

	K167N (rs11053646)		
	KK	KN+NN	p-value
Systolic blood pressure (mmHg)	135.830+/−0.630	133.39+/−1.47	ns
Diastolic blood pressure (mmHg)	84.42+/−0.34	83.17+/−0.91	ns
IMTm (mm)	0.663+/−0.004	0.694+/−0.011	0.050*

To next investigate how the presence of the LOX-1 NN genotype could be associated with an increased IMT, we performed a series on in vitro analysis in monocytes and macrophages from carriers of the KK, the KN and the NN genotype.

**Figure 1:**
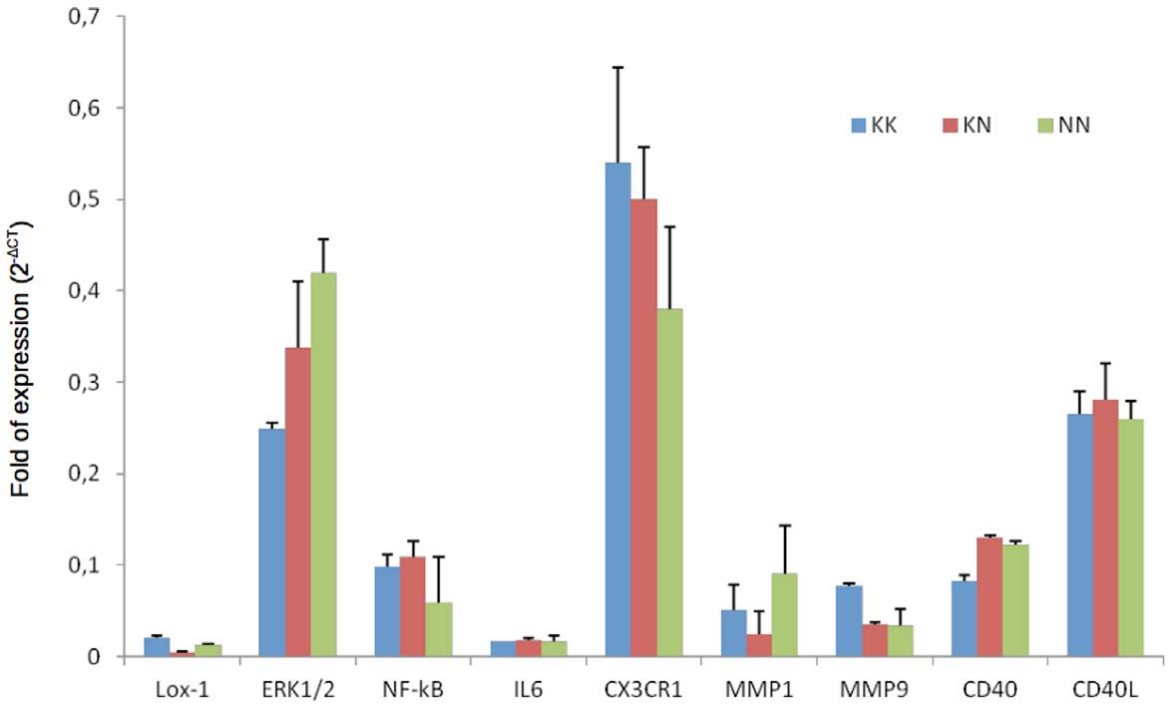
Gene expression levels from Peripheral Mononuclear Cells (PBMCs) of KK, KN and NN carriers. Expression levels are measured by the log (ΔΔCt) obtained comparing each gene's expression with that of the housekeeping gene, *HPRT1*. (*Nf-kB*: nuclear factor kappa-light-chain-enhancer of activated B cells, *ERK1/2*: extracellular related kinase 1/2, *IL-6*: Interleukin-6, *CD40*: cluster of designation 40, *CX3CR1*: CX3 chemokine receptor 1, *TLR-4*: Toll-like receptor 4, *MMP*: metalloproteinase).

**Figure 2:**
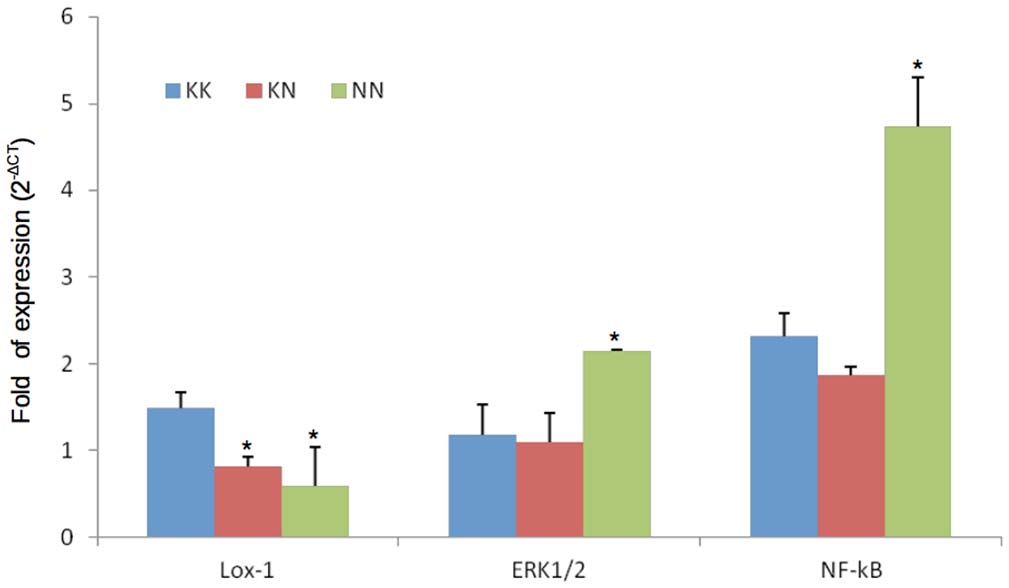
*OLR1* expression in KK, KN and NN in differentiated macrophages. (*Nf-kB*: nuclear factor kappa-light-chain-enhancer of activated B cells, *ERK1/2*: extracellular related kinase 1) (* p<0.05 vs KK).

Under basal conditions, as expected, *OLR1* expression was very low in PBMCs. The same was true for *Nf-kB* (nuclear factor kappa-light-chain-enhancer of activated B cells), *ERK1/2* (extracellular related kinase 1/2), *IL-6* (Interleukin-6), *CD40* (cluster of designation 40), *CD40* ligand, and *MMP9*. The mRNA expression of all tested genes in PBMCs from KK, KN and NN subjects was similar (Figure 1, Table S2). Next, to investigate LOX-1 NN, LOX-1 KN and LOX-1 KK effects in the proper atherosclerotic-prone setting, monocyte were differentiated into macrophages and gene expression analysis was performed. *Nf-kB* mRNA expression was significantly increased in NN compared to KN and KK macrophages (Figure 2 and Table S3). The same trend was observed for *ERK1/2*, although this difference did not reach a statistical significance (Figure 2 and Table S3). On the contrary, *OLR1* RNA expression was significantly lower in NN and KN macrophages (Figure 2 and Table S3) compared to KK macrophages.

**Figure 3:**
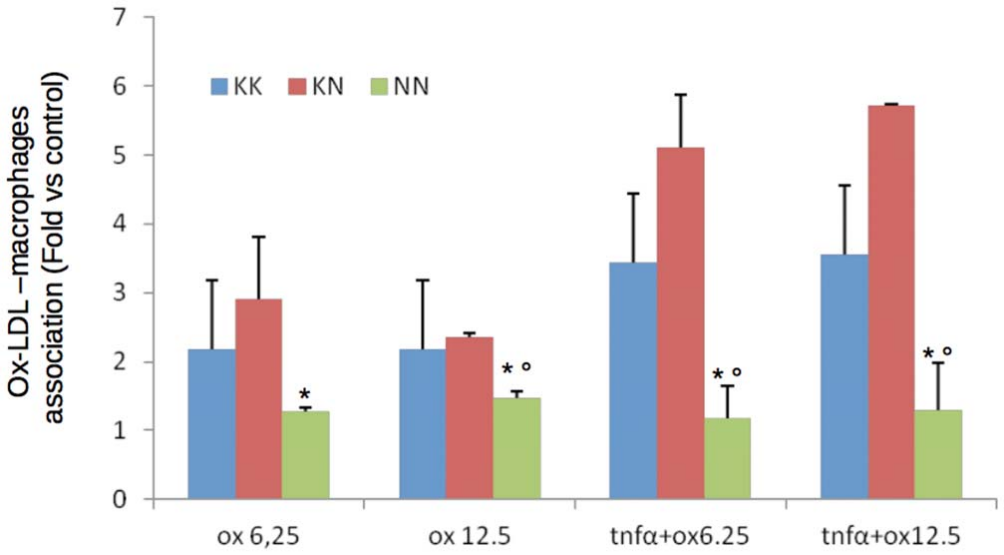
Association levels of OxLDL to macrophages from different genotypes incubated for 1 hours with 6.25 µg/mL DiO-Ox-LDL (A), 12.5 µg/mL DiO-OxLDL (B), 6.25 µg/mL DiO-OxLDL+ 10 ng/mL TNFα (C), 12.5 µg/mL DiO-OxLDL+ 10 ng/mL TNFα (D) (* p<0.05 vs KK; ° p<0.05 vs KN).

Finally we tested whether different LOX-1 genotypes might affect the association to Ox-LDL. Ox-LDL associate to NN macrophages to a lower extent compared to KN and KK macrophages (Fig. 3, Table S3). This result is agreement with previous findings in an African green monkey cell line (COS-1) (9). This was true when cells were incubated in the presence of Ox-LDL with or without LOX-1 induction obtained following incubation with TNFα (Figure 3, Table S4).

To summarize results, we identified the LOX-1 *167N* (C) allele as associated to higher level to IMT in males prior to any evidence of disease, we confirmed *in vivo* that NN macrophages internalize Ox-LDL to a lower extent and found evidences suggestive for an immune reaction to circulating Ox-LDL in early phases of the disease.

## Discussion

The main finding of this paper is that males from the general population with the C (N) allele of the *ORL1* gene have an increased IMT. Since no other phenotype resulted associated to this variant, this suggests it to be an independent association. To date, inconclusive results on the epidemiology of *OLR1* gene polymorphisms were obtained. Both a protective role for MI and CAD severity [10], [12], [14] and a risk role for MI and hypertension [11], [15] for the C (N) allele were proposed. Also, the replication of these results was rarely tested and never obtained [14], leading to the conclusion that these were spurious associations. Furthermore, a recent meta-analysis considered all studies that have been published on the relationship between *OLR1* polymorphisms and MI, finding partially contrasting results and an overall effect that is non-significant when considering the K167N variation [33].

Recently, a study reported the association between the C (N) allele and carotid plaque prevalence in 167 women from a Dominican-Hispanic population [16]. We extend these findings, showing an allele's association in 926 males from the PLIC population and analysing the molecular players and pathological responses associated with leucocytes and macrophages from NN, KN and KK subjects.

Previous data on COS-1 cells over expressing either GG (KK) or CC (NN) LOX-1 showed that KK COS-1 cells bind and internalize less ox-LDL than CC (NN) [7]. Here for the first time to our knowledge, we addressed this question in macrophages derived from peripheral mononuclear cells from KK and NN subjects, after stimulation with Ox-LDL and TNFα. We would expect that C (N) allele of the *ORL1* gene to be a protective factor, especially in the late phases of the disease. However, past studies demonstrated that circulating Ox-LDL trigger immune response and are associated to an increased IMT [17], [18], [19]. This suggests that, in non-pathological conditions, a decrease of macrophage-Ox-LDL association, and hence an increase of circulating Ox-LDL, may set the stage to trigger an immune response.

We report evidence that macrophages carrying the functional GG (KK) receptor display higher levels of *OLR1*'s RNA, but lower RNA expression levels of an inflammatory marker such as *Nf-kB*, suggesting again that, in non-pathological conditions, a less-functional CC (NN) receptor, could be associated to an increased inflammation.

Regarding the difference between genders of our study and that of Wang and co-workers, there are several aspects that have to be taken into account: First, we studied a completely different population. It is known, in fact, that Hispanics have different susceptibility to cardiovascular disease, only partially explainable by environmental factors [34]. Furthermore *OLR1* variants display highly different frequencies in different populations (p<0.0001 comparing European ancestry HapMap populations and African HapMap populations, and p = 0.04 comparing European ancestry populations and Mexicans in HapMap populations. Details about analyses on HapMap samples are available upon request) Second, gene-regulation for many quantitative traits differs significantly between males and females. In particular, evidences suggested a differential genotype by sex interaction on variation on Paraoxonase-1 (*PON1*, a calcium dependent esterase known to have antioxidant properties) activity in Mexican American populations [35]. We tested for interaction between available functional variants on both *OLR1* and *PON1* from the HapMap dataset and found suggestive evidences for differences in gene-by-gender interactions in populations with different ancestry (details about analyses on HapMap samples are available upon request). Third, Wang and co-workers focused their analysis on the presence of plaque, (advanced atherosclerosis) while we focused on IMT (atherogenesis) thus suggesting a dual role for *OLR1* in different phases of the atherogenic process.

Although we have to recognize some limits of our study, including the low number of NN individuals, this is the first study which was able to directly carry on functional tests on human NN PBMCs and macrophages and represents, to date, the second largest study which investigated the role of LOX-1 functional polymorphism in the onset of cardiovascular disorders. Therefore, these findings support the relevance of *OLR1* in vascular disorders at epidemiological and functional level.

## Supporting Information

Methods S1 **Sequencing of samples: All of the CC (NN) and ∼50 of the CG (KN) and GG (KK) genotypes were verified through sequencing using forward primer: ATGCACGTGAGAGAACTAAGGG and reverse primer: TGGCTCTCAAACAAGAATTCC (Applied Biosystems, Foster City, CA).** Two CC individuals turned out to be CG, but since for Statistical Analyses KK and KN were considered as a single group, results were not affected. All GG and CG individuals were confirmed.
(DOC)

Table S1 **Primer sequences for Gene Expression Analysis ((Nf-kB: nuclear factor kappa-light-chain-enhancer of activated B cells, ERK1/2: extracellular related kinase 1/2, IL-6: Interleukin-6, CD40: cluster of designation 40, CX3CR1: CX3 chemokine receptor 1, TLR-4: Toll-like receptor 4, MMP: metalloproteinase).**
(DOC)

Table S2 **Gene expression levels in PBMCs obtained from KK and NN. (** ***Nf-kB*** **: nuclear factor kappa-light-chain-enhancer of activated B cells,** ***ERK1/2*** **: extracellular related kinase 1/2,** ***IL-6*** **: Interleukin-6,** ***CD40*** **: cluster of designation 40,** ***CX3CR1*** **: CX3 chemokine receptor 1,** ***TLR-4*** **: Toll-like receptor 4,** ***MMP*** **: metalloproteinase).**
(DOC)

Table S3 ***OLR1*** **,** ***NF-kB*** **and** ***ERK1/2*** **expression levels in differentiated macrophages obtained from KK and NN PBMCs.** (*Nf-kB*: nuclear factor kappa-light-chain-enhancer of activated B cells, *ERK1/2*: extracellular related kinase 1/2).
(DOC)

Table S4 **OxLDL association levels to macrophages obtained from the different genotypes.**
(DOC)
