# Supplementary material for: Association between OLR1 K167N SNP and Intima Media Thickness of the Common Carotid Artery in the General Population
Source: PLoS One. 2012 Feb 9;7(2):e31086. doi: 10.1371/journal.pone.0031086 (PMC3276570; doi:10.1371/journal.pone.0031086)
Supplement: Methods S1 — Sequencing of samples: All of the CC (NN) and ∼50 of the CG (KN) and GG (KK) genotypes were verified through sequencing using forward primer: ATGCACGTGAGAGAACTAAGGG and reverse primer: TGGCTCTCAAACAAGAATTCC (Applied Biosystems, Foster City, CA). Two CC individuals turned out to be CG, but since for Statistical Analyses KK and KN were considered as a single group, results were not affected. All GG and CG individuals were confirmed. (DOC) [file pone.0031086.s001.doc]

SUPPLEMENTAL METHODS:

**Sequencing of samples:**

All of the CC (NN) and ~ 50 of the CG (KN) and GG (KK) genotypes were verified through sequencing using forward primer: ATGCACGTGAGAGAACTAAGGG and reverse primer: TGGCTCTCAAACAAGAATTCC (Applied Biosystems, Foster City, CA). Two CC individuals turned out to be CG, but since for Statistical Analyses KK and KN were considered as a single group, results were not affected. All GG and CG individuals were confirmed.
