# Supplementary material for: Association between OLR1 K167N SNP and Intima Media Thickness of the Common Carotid Artery in the General Population
Source: PLoS One. 2012 Feb 9;7(2):e31086. doi: 10.1371/journal.pone.0031086 (PMC3276570; doi:10.1371/journal.pone.0031086)
Supplement: Table S1 — Primer sequences for Gene Expression Analysis ((Nf-kB: nuclear factor kappa-light-chain-enhancer of activated B cells, ERK1/2: extracellular related kinase 1/2, IL-6: Interleukin-6, CD40: cluster of designation 40, CX3CR1: CX3 chemokine receptor 1, TLR-4: Toll-like receptor 4, MMP: metalloproteinase). (DOC) [file pone.0031086.s002.doc]

**Supplemental Table S**1. Primer sequences for Gene Expression Analysis ((Nf-kB: nuclear factor kappa-light-chain-enhancer of activated B cells, ERK1/2: extracellular related kinase 1/2, IL-6: Interleukin-6, CD40: cluster of designation 40, CX3CR1: CX3 chemokine receptor 1, TLR-4: Toll-like receptor 4, MMP: metalloproteinase)

| **GENE** | **PRIMER FORWARD** | **PRIMER REVERSE** |
| --- | --- | --- |
| ***HPRT1*** | TTATGGACAGGACTGAACGTCTTG | AGCAGGTCAGCAAAGATT |
| ***OLR1*** | GCACAGCTGATCTGGACTTCAT | CCCCATCCAGAATGGAAAACT |
| ***ERK1/2*** | CCATCGAGCAAATGAAAGATGTATATAT | CTGAGGATCTGGTAGAGAAAATAGCA |
| ***NF-kB*** | AAAGGTTATTGTTCAGTTGGTCACAA | GCGAAGCCGACCACCAT |
| ***IL-6*** | CCAGGAGCCCAGCTATGAAC | CCCAGGGAGAAGGCAACTG |
| ***CX3CR1*** | TCTCACCTCGCTATGGTTCGT | GCTGGACAGCGGTCAGCAA |
| ***TLR-4*** | AATTCCATGGTATTATTGA | TCTTTTACCCTTTCAATAGT |
| ***MMP-1*** | AAACACATCTGACCTACAGGATTGAA | GGCTTTCTCAATGGCATGGT |
| ***MMP-9*** | CCGGACCAAGGATACAGTTTG | TGAAGCGGTACATAGGGTACATGA |
| ***CD40*** | TCTCACCTCGCTATGGTTCGT | GCTGGACAGCGGTCAGCAA |
| ***CD40 ligand*** | CCCCCGGTAGATTCGAGAGA | TGCCCGCAAGGTTTGG |
