# Supplementary material for: Association between OLR1 K167N SNP and Intima Media Thickness of the Common Carotid Artery in the General Population
Source: PLoS One. 2012 Feb 9;7(2):e31086. doi: 10.1371/journal.pone.0031086 (PMC3276570; doi:10.1371/journal.pone.0031086)
Supplement: Table S2 — Gene expression levels in PBMCs obtained from KK and NN. ( Nf-kB : nuclear factor kappa-light-chain-enhancer of activated B cells, ERK1/2 : extracellular related kinase 1/2, IL-6 : Interleukin-6, CD40 : cluster of designation 40, CX3CR1 : CX3 chemokine receptor 1, TLR-4 : Toll-like receptor 4, MMP : metalloproteinase). (DOC) [file pone.0031086.s003.doc]

**Supplemental Table S2. Gene expression levels in PBMCs obtained from KK and NN. (*Nf-kB*: nuclear factor kappa-light-chain-enhancer of activated B cells, *ERK1/2*: extracellular related kinase 1/2, *IL-6*: Interleukin-6, *CD40*: cluster of designation 40, *CX3CR1*: CX3 chemokine receptor 1, *TLR-4*: Toll-like receptor 4, *MMP***: metalloproteinase).

|  | **KK** | **KN** | **NN** | **P value.** |
| --- | --- | --- | --- | --- |
| ***OLR1*** | 0.020+/-0.003 | 0.004+/-0.003 | 0.013+/-0.000 | 0.88 |
| ***ERK1/2*** | 0.249+/-0.007 | 0.338+/-0.072 | 0.420+/-0.037 | 0.07 |
| ***NF-kB*** | 0.098+/-0.013 | 0.109+/-0.017 | 0.058+/-0.051 | 0.17 |
| ***IL-6*** | 0.016+/-0.001 | 0.017+/-0.003 | 0.017+/-0.006 | 0.21 |
| ***CX3CR1*** | 0.540+/-0.104 | 0.501+/-0.056 | 0.380+/-0.090 | 0.19 |
| ***MMP-1*** | 0.051+/-0.027 | 0.024+/-0.025 | 0.090+/-0.052 | 0.21 |
| ***MMP-9*** | 0.077+/-0.003 | 0.034+/-0.003 | 0.033+/-0.018 | 0.19 |
| ***CD40*** | 0.083+/-0.002 | 0.130+/-0.002 | 0.122+/-0.004 | 0.24 |
| ***CD40 ligand*** | 0.264+/-0.026 | 0.281+/-0.040 | 0.259+/-0.020 | 0.21 |
