# Supplementary material for: Association between OLR1 K167N SNP and Intima Media Thickness of the Common Carotid Artery in the General Population
Source: PLoS One. 2012 Feb 9;7(2):e31086. doi: 10.1371/journal.pone.0031086 (PMC3276570; doi:10.1371/journal.pone.0031086)
Supplement: Table S3 — OLR1 , NF-kB and ERK1/2 expression levels in differentiated macrophages obtained from KK and NN PBMCs. (Nf-kB: nuclear factor kappa-light-chain-enhancer of activated B cells, ERK1/2: extracellular related kinase 1/2). (DOC) [file pone.0031086.s004.doc]

**Supplemental Table S3. *OLR1,* *NF-kB* and *ERK1/2* expression levels in differentiated macrophages obtained from KK and NN PBMCs. (*Nf-kB*: nuclear factor kappa-light-chain-enhancer of activated B cells, *ERK1/2***: extracellular related kinase 1/2)

|  | **KK** | **KN** | **NN** | **P value KK vs NN** | **P value KN vs NN** |
| --- | --- | --- | --- | --- | --- |
| ***OLR1*** | 1.484+/-0.183 | 0.814+/-0.359 | 0.583+/-0.264 | 0.015 | 0.0002 |
| ***NF-kB*** | 2.324+/-0.456 | 1.872+/-0.012 | 4.741+/-0.559 | 0.123 | 0.028 |
| ***ERK1/2*** | 1.177+/-0.114 | 1.091+/-0.337 | 2.145+/-0.091 | 0.696 | 0.167 |
