# Supplementary material for: Association between OLR1 K167N SNP and Intima Media Thickness of the Common Carotid Artery in the General Population
Source: PLoS One. 2012 Feb 9;7(2):e31086. doi: 10.1371/journal.pone.0031086 (PMC3276570; doi:10.1371/journal.pone.0031086)
Supplement: Table S4 — OxLDL association levels to macrophages obtained from the different genotypes. (DOC) [file pone.0031086.s005.doc]

**Supplemental Table S**4. OxLDL association levels to macrophages obtained from the different genotypes.

| **Cell Treatment** | **KK** | **KN** | **NN** | **P value KK vs NN** | **P value KN vs NN** |
| --- | --- | --- | --- | --- | --- |
| Ox-LDL 6.25 | 2.176+/-0.142 | 2.899+/-0.915 | 1.267+/-0.065 | 0.255 | 0.002 |
| Ox-LDL 12.5 | 2.178+/-0.114 | 2.346+/-0.058 | 1.473+/-0.098 | 0.005 | 0.005 |
| TNFα + Ox-LDL 6.25 | 3.445+/-0.569 | 5.102+/-0.780 | 1.17+/-0.473 | 0.027 | 0.006 |
| TNFα + Ox-LDL 12.5 | 3.552+/-0.490 | 5.710+/-0.022 | 1.301+/-0.681 | 0.017 | 0.000 |
